# Judging Older Adult Speakers: Only Young Adults Low in Ageism Notice the Nuances

**DOI:** 10.1093/geroni/igaa057.3223

**Published:** 2020-12-16

**Authors:** Jennifer Turner, Renee Hayslip, Jennifer Stanley

**Affiliations:** 1 Pennsylvania State University, State College, Pennsylvania, United States; 2 University of Akron, Akron, Ohio, United States

## Abstract

Ageism negatively impacts hiring and electability success, as well as intergenerational relationships (Levy,2003;2009). The current study sought to examine whether personality cues influenced performance ratings of older adult (OA) speakers whose behavior had been modified by an embodiment intervention (i.e., “power posing”). One-hundred-and-three young adults (YA; Mage=19.6, SD=2.06; 49.5% women) rated the performance of 9 OA speakers performing 5-minute campaign speeches, and reported the cues that influenced their ratings. Two independent raters coded the cues (i.e., introversion and extroversion; coded by two independent raters, κ = .72 [moderate-to-substantial interrater reliability; Chen, 2019; McHugh, 2012]). Participants also completed the Refined-Aging Semantic Differential (Polizzi & Millikin, 2002) as a measure of ageism endorsement. Greater ageism was associated with lower performance ratings (F(1,101)=15.97, p<.001, R2=.14); performance was reduced by .12, 95%CIs[-.018,-.006] for each additional point of ageism endorsement. Next, we investigated whether personality cues would modify this relationship using Hayes PROCESS Model 1 (2018). A significant interaction emerged between ageism and introverted cues (b=.015, p=.05, 95%CIs[.006,.023]), suggesting that greater perceived introversion was negatively associated with performance ratings. Additionally, individuals lower in ageism were more likely to calibrate their performance judgments based on the pose condition of the speaker, with participants lower in ageism exhibiting greater variability across pose conditions (Ms=3.96-4.37), than individuals higher in ageism (Ms=4.21-4.31), suggesting that individuals lower in ageism were attending to nuances in the speech (e.g., pose influence), while higher ageist individuals relied more heavily on age-related judgments.

